# Mutations in many genes affect aggressive behavior in *Drosophila melanogaster*

**DOI:** 10.1186/1741-7007-7-29

**Published:** 2009-06-11

**Authors:** Alexis C Edwards, Liesbeth Zwarts, Akihiko Yamamoto, Patrick Callaerts, Trudy FC Mackay

**Affiliations:** 1 Department of Genetics, North Carolina State University, Raleigh, NC, USA; 2 W. M. Keck Center for Behavioral Biology, North Carolina State University, Raleigh, NC, USA; 3 Department of Biology, North Carolina State University, Raleigh, NC, USA; 4 Laboratory of Developmental Genetics, VIB-PRJ8 & Katholieke Universiteit Leuven, Center for Human Genetics, Leuven, Belgium; 5 Current address: Virginia Institute for Psychiatric and Behavioral Genetics, Virginia Commonwealth University, Department of Psychiatry, Richmond, VA, USA

## Abstract

**Background:**

Aggressive behavior in animals is important for survival and reproduction. Identifying the underlying genes and environmental contexts that affect aggressive behavior is important for understanding the evolutionary forces that maintain variation for aggressive behavior in natural populations, and to develop therapeutic interventions to modulate extreme levels of aggressive behavior in humans. While the role of neurotransmitters and a few other molecules in mediating and modulating levels of aggression is well established, it is likely that many additional genetic pathways remain undiscovered. *Drosophila melanogaster *has recently been established as an excellent model organism for studying the genetic basis of aggressive behavior. Here, we present the results of a screen of 170 *Drosophila P*-element insertional mutations for quantitative differences in aggressive behavior from their co-isogenic control line.

**Results:**

We identified 59 mutations in 57 genes that affect aggressive behavior, none of which had been previously implicated to affect aggression. Thirty-two of these mutants exhibited increased aggression, while 27 lines were less aggressive than the control. Many of the genes affect the development and function of the nervous system, and are thus plausibly relevant to the execution of complex behaviors. Others affect basic cellular and metabolic processes, or are mutations in computationally predicted genes for which aggressive behavior is the first biological annotation. Most of the mutations had pleiotropic effects on other complex traits. We characterized nine of these mutations in greater detail by assessing transcript levels throughout development, morphological changes in the mushroom bodies, and restoration of control levels of aggression in revertant alleles. All of the *P*-element insertions affected the tagged genes, and had pleiotropic effects on brain morphology.

**Conclusion:**

This study reveals that many more genes than previously suspected affect aggressive behavior, and that these genes have widespread pleiotropic effects. Given the conservation of aggressive behavior among different animal species, these are novel candidate genes for future study in other animals, including humans.

## Background

Aggressive behavior in animals is important for survival and reproduction. Aggression is used for self-defense against con-specifics and predators, in acquisition of territory, food and mates, and in defense of progeny. However, aggressive behaviors are energetically expensive, and there is likely an intermediate optimum level of aggression in natural populations from a balance between the energy and risk associated with territory defense and the need to find food and mates. In social organisms such as humans or other primates, an extremely high level of aggression can be disadvantageous or even pathological.

Aggressive behaviors are quantitative traits, with continuous variation in natural populations due to segregating alleles at multiple interacting loci, with effects that are sensitive to developmental and environmental conditions. Identifying the underlying genes and environmental contexts that affect aggressive behavior is necessary if we are to understand the evolutionary forces acting to maintain variation for aggressive behavior in natural populations, and to develop therapeutic interventions to modulate extreme levels of aggressive behavior in humans. Much of the work on the neurobiology and genetics of aggressive behavior to date has used the candidate gene approach to establish the role of neurotransmitters in mediating and modulating levels of aggression. In particular, biogenic amines and genes affecting their biosynthesis and metabolism have been associated with aggressive behavior in mammals [1-7] and invertebrates [8-15]. The neurotransmitters nitric oxide and γ-aminobutyric acid also modulate aggressive behavior in mammals [15-17]. Neuropeptide Y affects aggression in mammals [18-20] and its invertebrate homolog, neuropeptide F, affects aggression in *Drosophila *[13]. In *Drosophila*, correct expression of the male-specific transcript of *fruitless*, a gene in the sex-determination pathway, is required for executing male aggressive behaviors [12,21-23].

*Drosophila *exhibit territorial behavior in wild populations [24,25], and therefore represent an excellent model system for investigating the genetic basis of aggressive behavior. Recent studies have revealed a much more complex genetic architecture of *Drosophila *aggression than suggested by targeted evaluation of candidate genes in biologically plausible pathways. Many novel loci affecting aggressive behavior have been implicated from widespread correlated responses in gene expression to selection for divergent levels of aggressive behavior [26,27]. Subsequent evaluation of aggressive behavior of mutations in a sample of these candidate genes revealed that a large number indeed affected aggressive behavior, including mutations in a member of the cytochrome P450 gene family [26]; and genes involved in electron transport, catabolism, nervous system development, G-protein coupled receptor signaling, as well as computationally predicted genes [27,28]. Analysis of quantitative trait loci (QTLs) affecting variation in aggression between two wild-type strains also identified a complex genetic basis for natural variation in aggressive behavior, characterized by extensive epistasis among QTLs [29]. Complementation tests to mutations at positional candidate genes in the QTL regions also revealed four additional novel loci affecting aggressive behavior [29]. These results motivate a broader screen for mutations affecting *Drosophila *aggression.

Previously, we developed a highly reproducible and rapid assay to quantify levels of aggression in *D. melanogaster *males [27]. Here, we employed a modified version of this assay to screen 170 *P{GT1} *transposable element (*P*-element) mutant lines that were generated in the same co-isogenic background. All of these lines are viable and fertile as homozygotes; therefore, the mutations are unlikely to be genetic null alleles. This is obviously an essential criterion for evaluating effects of mutations in essential genes on behavioral traits expressed in adults, and the quantitative assay enables detection of mutations with subtle as well as large effects. Further, the exact insertion site of the transposon, and thus the identity of the candidate gene(s) it disrupts, can be readily determined. The same panel of lines has been screened for mutations affecting numbers of sensory bristles [30], resistance to starvation stress [31], sleep [32] and olfactory [33] and locomotor [34] behavior, enabling us to assess pleiotropic mutational effects. We identified 59 mutations in 57 genes that affect aggressive behavior, none of which had been previously implicated to affect aggression. While many of the genes affect the development and function of the nervous system, and are thus plausibly relevant to the execution of complex behaviors, others affect basic cellular and metabolic processes, or computationally predicted genes for which aggressive behavior is the first biological annotation. Most of the mutations had pleiotropic effects on other complex traits. More detailed characterization of nine of the mutations indicated that the *P*-element insertions affected the tagged genes, and that the mutations had pleiotropic effects on brain morphology.

## Methods

### Drosophila stocks

Flies were reared on cornmeal/molasses/agar medium under standard culture conditions (25°C, 12:12 hour light/dark cycle). CO_2 _was used as an anesthetic. All mutant lines are homozygous and contain single *P{GT1} *transposable element inserts in the *w*^1118 ^*Canton-S B *co-isogenic background, and were constructed as part of the Berkeley Drosophila Gene Disruption Project [35]. Male *w*^1118 ^*Canton-S B *flies were used as the control line.

### Quantitative assay for aggressive behavior

Assays were performed on socially experienced, 3–7 day-old male flies. Groups of eight males from the same mutant line were anesthetized 24 hours prior to the assay and placed in vials with food. On the day of the assay, the males were transferred without anesthesia to an empty vial and were deprived of food for 90 minutes, after which they were exposed to a food droplet and given one minute to acclimate to this disturbance. The flies were then observed for an additional one minute, and the total number of aggressive encounters scored as described previously [27]. Behavioral assays were conducted in a behavioral chamber (25°C, 70% humidity) between 8 a.m. and 11 a.m.

The screen was conducted in 34 blocks of 1–7 mutant lines and the contemporaneous control, with 20 replicate vials for each mutant line and the control line per block. *P*-element insert lines with significantly different levels of aggression than the control were identified using a one-way fixed effect ANOVA model. *Post-hoc *Tukey tests were used to determine whether aggression levels of mutant lines in a block differed significantly from that of the control after correcting for multiple tests. In addition, a one-way random effects ANOVA was performed on the entire data set, expressing the aggressive behavior of the mutant lines as deviations from their contemporaneous control. The among line (σ_*L*_^2^) and within line (σ_*E*_^2^) variance components were computed, and the broad sense mutational heritability estimated as:



### Bioinformatics

Gene ontology categories among *P*-element insert lines associated with increased or decreased levels of aggression were assessed using DAVID [36] and Babelomics [37]. Only gene ontology categories that applied to greater than 5% of the genes queried were considered in these analyses. Human orthologs of the genes tagged by the *P*-elements were assessed using FlyAtlas [38].

### Generation and verification of revertant lines

Genetic revertants were generated using standard crossing schemes, while preserving the co-isogenic background of the parental and revertant strains [32]. PCR products were sequenced to ascertain whether revertants were genetically precise. Primers were chosen to span either the 5' or 3' site of the original insertion. PCR products were run on 2% agarose gels and compared with a DNA ladder to determine whether they were of the appropriate length. Sequencing reactions were run on the PCR products, and the sequence of each *P *[-] line was compared with that of the control *w*^1118 ^*Canton-S B *to determine whether the excision of the *P*-element was precise.

### RNA extraction and cDNA generation

Samples were collected in triplicate from each of the following developmental stages: embryos aged 12–14 hours after egg laying (AEL); third instar larvae; pupae aged 8–9 days AEL; and male adults aged 3–5 days post-eclosion, with heads and bodies separated. Whole RNA was extracted using Trizol (GIBCO-BRL, Gaithersburg, MD) and purified using standard procedures. cDNA was generated using 500 ng of whole RNA with reagents from Applied Biosystems (Foster City, CA).

### Quantitative reverse transcription-PCR

Primers for quantitative reverse transcription PCR (qPCR) were obtained from Sigma (St. Louis, MO) and targeted gene regions common to all transcripts. cDNA was diluted 1:6 for a concentration of 41.7 ng/μl, and 2 μl cDNA were used for each 10 μl qPCR reaction. Each biological replicate was assessed in three technical replicates. An ABI-7900 sequence detector and protocols from Applied Biosystems were used to perform the qPCR with the SYBR Green detection method. Relative mRNA quantities were standardized using the housekeeping gene *Glyceraldehyde-3-phosphate dehydrogenase 1 *(*Gapdh1*). Standardization was conducted on *Ct *values reported by the ABI-7900 software. Since *Ct *values are relative exponential measures, standardized values were converted to linearized values as described in Livak and Schmittgen [39] for statistical tests. Differences in gene expression level between the *P*-element insert line and the control line were tested for statistical significance using two-tailed Student's *t*-tests.

### Whole-mount in situ hybridization

cDNA clones for *ed *(LD11008), *sgl *(SD09476), *emc *(LD10532), *pbx *(RE16319), *Syx4 *(RE02884), *CG13377 *(RE15974), *CG32572 *(AT02481) and *CG3638 *(LD20542) were ordered from the Berkeley Drosophila Genome Project. *Act5c *cDNA was obtained using the High Fidelity PCR System (Roche Applied Science, Indianapolis, IN) on *Canton-S *genomic DNA using the following primers: 5' ATGTGTGACGAAGAAGTTGCTG, 5' CACGTGGCGTTCACGAAGATT. The 1131 bp fragment was cloned into the *pCR^®^II-TOPO *vector (Invitrogen). Digoxigenin-labeled sense and antisense RNA probes were synthesized by *in vitro *transcription using the DIG RNA labeling kit (Roche Applied Science). The probes were hydrolyzed at 60°C in buffer containing 200 mM Na_2_CO_3 _and 200 mM NaHCO_3 _(*X *= (*Lo *- *Ld*)/(0.11 × *Lo *× *Ld*) with *Lo *= original length of the transcript in kb; *Ld *= desired 0.2 kb length), precipitated in ethanol and resuspended in RNase-free water. Whole-mount *in situ *hybridization was performed using a variation of the protocol described by Tautz and Pfeifle [40]. Signal detection was carried out using anti-digoxigenin-AP Fab fragments (Roche Applied Science). Color development was performed in the dark using 0.5 mg/ml NBT (Roche Applied Science) and 0.25 mg/ml BCIP (Roche Applied Science). Embryos were 0–17 h old. Images were obtained using a light microscope (model BX61; Olympus) and Cell^D 2.6 imaging software.

### Immunohistochemistry and morphometric analysis

Immunohistochemical labeling of adult *Drosophila *brains with anti-fasciclin II MAb 1D4 (Developmental Studies Hybridoma Bank; under the auspices of the NICHD and maintained by the University of Iowa, Department of Biological Sciences, Iowa City, IA 52242) and morphometric analyses of mushroom body lobes and ellipsoid body were done as previously described [28]. Measurements were taken for each hemisphere of 10 brains per line. Statistical significance was determined using *t*-tests for differences between mutant lines and the *Canton-S B *control line.

## Results and discussion

### Mutations affecting aggressive behavior

We quantified aggressive behavior of *P*-element insertional mutations that had been generated in a common isogenic background (*Canton S B *[35]), as well as the control line (Additional file 1). The 170 *P*-element lines represented insertions in 148 genes. Approximately one-half of these lines were chosen because they represent mutations in genes that exhibited changes in transcript abundance as a correlated response to artificial selection for aggressive behavior [27]. We also included genes if they had previously been shown to have a mutant phenotype for a different behavior, to examine possible pleiotropic effects of mutations on aggression and other behaviors; or if the *P*-element tagged a computationally predicted gene, to provide potential biological annotations for these sequences.

**Figure 1:**
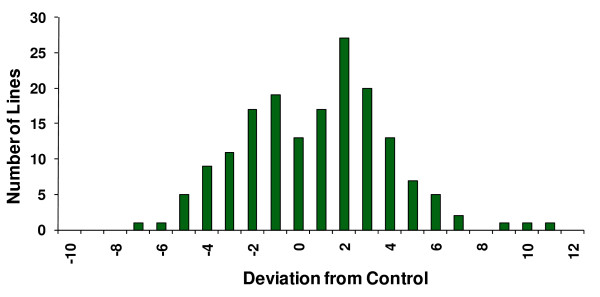
**Distribution of mean aggression scores among *170 P{GT1}-*element insertion lines, expressed as deviations from the co-isogenic *Canton-S B *control line**.

The broad sense mutational heritability (*H*_*M*_^2^) for aggressive behavior was rather high: *H*_*M*_^2 ^= 0.432. The high mutational heritability could be due to a few mutants of large effect, or many mutants with smaller effects. Analysis of the effects of individual mutations revealed that the latter was the case. A total of 59 (approximately 35%) of the *P*-element insert lines exhibited levels of aggression that differed significantly from the control; 27 lines were less aggressive, and 32 lines were more aggressive than the control line (Table 1, Figure 1). The absolute values of the standardized mutational effects (*a*/*σ*, where *a *is one-half of the difference in the mean aggression score of the *P*-element insert line and the control line, and *σ *is the phenotypic standard deviation of the control line) of the 59 lines with significantly increased or decreased levels of aggression ranged from 0.28 to 2.27, with a mean of 0.77 (Table 1).

**Table 1:** *P{GT1} *lines with aberrant aggressive behavior

***P*-element line**	**Disrupted locus**	**Cytogenetic location**	**Mutational effects**		**Pleiotropic effects**					**Gene ontology**
				
			**MAS**	***a/σ*_*P*_**	**B**	**L**	**O**	**Sl**	**St**	
BG00151	*CG9894*	23A3–23A3	16.35**	0.96						Unknown
BG00336	*Guanine nucleotide exchange factor GEF64C*	64B13–64B17	6.1***	-1.45					↓	Axon guidance; regulation of Rho protein signal transduction
BG00372	*CG1678*	20A1-20A1	17.2***	0.94				↑		Unknown
BG00375	*Odorant-binding protein 99d*	99B8-99B8	14.8**	0.53	↑				↓	Odorant binding; autophagic cell death; transport
BG00376†	*CG3638*	1E4-1E4	18.45***	1.16	↑			↑	↓	Unknown
BG00386	*NMDA receptor 1*	83A6–83A7	20.65***	1.53					↓	Long-term memory; olfactory learning; calcium-mediated signaling; nerve-nerve synaptic transmission; nervous system development
BG00670	*CG32541*	17F3-18A2	13.35**	0.28	↑					Unknown
BG00735	*schizo*	78A5-78B1	16.25***	0.78					↑	Central nervous system development; guanyl-nucleotide exchange factor activity
BG00986†	*extra macrochaetae*	61C9-61C9	7.05***	-0.90	↑	↓		↑	↓	Nervous system development
BG01011	*Spinophilin*	62E4–62E5	17.2***	0.69		↓		↑	↑	Phosphopantetheine binding
BG01043	*Gp150*	58D3-58D3	18.0*	0.91						ATP binding; transmembrane receptor protein tyrosine phosphatase signaling pathway
BG01046	*CG3587*	2B16-2B16	7.9***	-0.60	↑	↓			↓	Unknown
BG01130	*alan shepard*	64C8–64C11	16.55***	0.86				↑	↓	mRNA processing; gravitaxis
BG01214†	*sugarless*	65D4–65D5	17.1***	0.93				↑	↓	Cell communication; signal transduction; transmembrane receptor protein tyrosine kinase signaling pathway
BG01215	*CG11299*	59F6–59F7	8.7**	-0.48	↑	↓			↓	Regulation of progression through cell cycle; cell cycle arrest
BG01299†	*Actin 5C*	5C7-5C7	3.6***	-1.75	↓	↓				structural constituent of cytoskeleton; ATP binding; protein binding
BG01354	*CG30492*	43E5–43E7	18.55**	1.01	↑					Zinc ion binding
BG01402	*CG32345*	61C7-61C7	15.55***	0.68		↓			↓	Unknown
BG01433	*CG13791*	28B1–28B2	16.75**	0.60		↓			↓	Unknown
BG01469†	*Syntaxin 4*	3B4-3B4	7.9***	-0.65		↓			↓	t-SNARE activity; neurotransmitter secretion; vesicle-mediated transport; synaptic vesicle docking during exocytosis
BG01491	*tramtrack*	100D1-100D1	14.45*	0.48	↑				↓	Zinc ion binding; peripheral nervous system development; transmission of nerve impulse
BG01498	*Casein kinase Iα*	11B7-11B7	8.85*	-0.43	↑				↓	Receptor signaling protein serine/threonine kinase activity; ATP binding
BG01536	*Beadex*	17C3–17C4	7.25***	-0.60	↑	↓			↓	Zinc ion binding; locomotory behavior; response to cocaine; regulation of metabolism
BG01566	*arrest*	33D3–33D5	6.75***	-0.75						Negative regulation of oskar mRNA translation
BG01596†	*CG13377*	1A1-1A1	19.55***	1.74		↓	↑	↑	↓	Metabolism
BG01654	*pickpocket 23*	16B4-16B4	9.55**	-0.57		↓			↑	Sodium channel activity
BG01662	*Laminin A*	65A8–65A9	15.55**	0.68				↑	↓	Receptor binding; locomotion; central nervous system development
BG01683†	*CG32572*	15A3-15A3	7.65***	-0.66		↓			↓	Unknown
BG01693	*CG10777*	7C3–7C4	4.5***	-1.11		↓	↑		↓	RNA helicase activity; nucleic acid binding; ATP binding; ATP-dependent helicase activity
BG01713	*4EHP*	95E1-95E1	8.9**	-0.50		↓				Translation initiation
BG01733	*CG6175*	68C1–68C2	14.55*	0.51	↑	↓			↓	Unknown
BG01757	*CG17323*	37B1-37B1	12.75*	0.50		↓				Defense response; polysaccharide metabolism; response to toxin; steroid metabolism
BG01765	*Tehao*	34C1-34C1	8.35**	-0.70	↓					Transmembrane receptor activity; signal transduction; Toll signaling pathway
BG01893	*Splicing factor 1*	90B4-90B4	7.2*	-0.51	↑	↓			↓	Transcription cofactor activity; nucleic acid binding; zinc ion binding
BG01900	*mir-317*	85F10-85F10	6.6**	-0.62	↑	↓			↓	microRNA
BG01909	*CG14035*	25C6-25C6	21.45***	2.27	↓	↓	↑		↓	Unknown
BG01912†	*pxb*	89A1–89A2	17.3***	0.85	↑	↓				Learning and/or memory; olfactory learning
BG01916	*no ocelli*	35B2-35B2	13.65**	0.36				↑	↓	Zinc ion binding
BG01949	*ade5*	11B16–11B16	16.2*	0.57					↓	Purine base metabolism; 'de novo' IMP biosynthesis
BG02019	*CG9171*	25F4–25F5	13.3**	0.60						Transferase activity
BG02022	*CG34460*	2R	7.15***	-0.91		↓	↑			Unknown
BG02077	*Rtnl1*	25B9-25C1	9.0*	-0.65	↑			↓	↓	Receptor signaling protein activity; calcium ion binding
BG02081	*Rtnl1*	25B9-25C1	16.6***	1.19		↓	↑			Receptor signaling protein activity; calcium ion binding
BG02095†	*echinoid*	24D4–24D6	14.8**	0.68					↑	Epidermal growth factor receptor signaling pathway; negative regulation of neurogenesis; sensory organ development
BG02128	*lethal (1) G0007*	12E3–12E5	5.55***	-0.86		↓			↓	ATP-dependent RNA helicase activity; ATP-dependent helicase activity; ATP binding
BG02188	*eclair*	85E4-85E4	15.8***	0.56	↑					Intracellular protein transport
BG02217	*plexus*	58E4–58E8	4.6***	-0.99	↓	↓				Wing vein morphogenesis
BG02276	*lethal (3) L1231*	88C10-88C10	10.95*	0.44	↑					Unknown
BG02377	*CG14478*	54B16-54B16	4.2**	-0.56	↓/↑					Unknown
BG02420	*CG5946*	68E1-68E1	3.85**	-0.61	↑					Cholesterol metabolism; electron transport; fatty acid desaturation
BG02470	*CG8963*	53E4-53E4	7.6*	-0.52	↓	↓	↑			Unknown
BG02495	*ade5*	11B16-11B16	13.35*	0.33				↑	↓	Purine base metabolism; 'de novo' IMP biosynthesis
BG02501	*longitudinals lacking*	47A11–47A13	7.9*	-0.61	↑					Axon guidance; axonogenesis; transmission of nerve impulse
BG02522	*CG42270*	16C1–16C8	6.1***	-0.80		↓	↑			Ras GTPase activator activity; receptor binding; G-protein coupled receptor protein signaling pathway;
BG02523	*lamina ancestor*	64C12–64C13	7.95***	-0.59	↑					Unknown
BG02539	*Basigin*	28E3–28E5	15.5**	-0.58				↑	↓	Spermatid development
BG02542	*neuralized*	85C2–85C3	11.35*	-0.60	↑					Ubiquitin-protein ligase activity; protein binding; zinc ion binding; nervous system development; sensory organ development; regulation of Notch signaling pathway
BG02644	*Fkbp13*	57E6-57E6	12.0**	0.59	↑					Calcium ion binding; protein folding
BG02731	*longitudinals lacking*	47A11–47A13	13.6**	0.63	↓					Axon guidance; axonogenesis; Transmission of nerve impulse

MAS = Mean Aggression Score. Significant MAS deviations from the control line after correcting for multiple tests are indicated by asterisks. *: *P *< 0.05; **: *P *< 0.01; ***: *P *< 0.0001. † Indicates lines characterized in greater detail. Pleiotropic effects of the mutations are given for numbers of sensory bristles (B) [30], locomotor startle response (L) [34]; olfactory avoidance behavior (O) [33]; male 24-hour sleep (Sl) [32] and male starvation stress resistance (St) [31]. The arrows indicate significant positive (↑) and negative (↓) deviations from the control.

The high proportion of mutations associated with alterations in aggressive behavior is likely in part to be because the screen was enriched for mutations in candidate genes previously implicated to affect aggression [27] and with mutations affecting other quantitative traits [30-34]. However, the large mutational target size for aggressive behavior is consistent with a growing body of evidence that large numbers of loci can affect most quantitative traits [27,30-34,41-46].

### Gene ontology analysis

**Figure 2:**
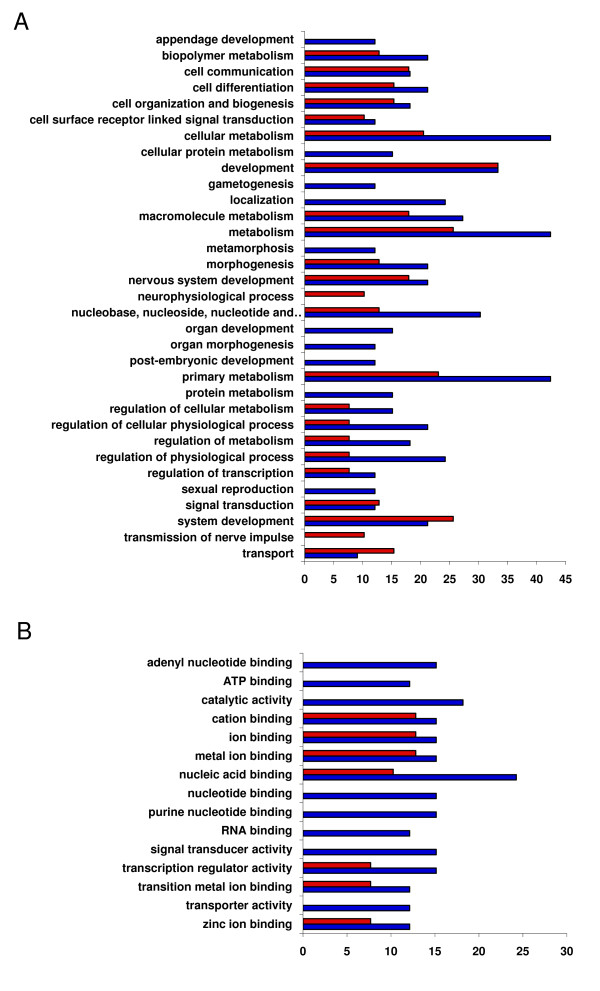
**Gene ontologies of candidate genes with mutations associated with aggressive behavior**. **(A) **Biological Process gene ontology categories. **(B) **Molecular Function gene ontology categories. The *x*-axis indicates the percentage of genes in each category for mutations increasing (red bars) and decreasing (blue bars) aggressive behavior.

The genes tagged by the *P*-element inserts associated with increased or decreased levels of aggression spanned a variety of gene ontology categories [36,37] (Figure 2). Many of these genes affect early development, including the development of the nervous system, and are involved in transcriptional regulation, signal transduction, and ATP binding. There is a trend towards differential representation of some gene ontology categories between the lines associated with increased versus decreased levels of aggression (Figure 2), although the differences are not significant due to the small numbers of mutations. For example, approximately 42% of the mutations with low levels of aggression are in genes affecting metabolism, but only approximately 26% of mutations with high levels of aggression fall into this category. A plausible interpretation is that dysfunction of metabolic processes can lead to a lower propensity to expend energy on demanding behaviors. Over 24% of mutations with low levels of aggression affect 'localization'; no mutations with high levels of aggression affect localization. The connection between this biological process and aggressive behavior is not intuitively obvious. Nearly all 59 genes tagged by *P*-elements that were associated with increased or decreased levels of aggression have orthologs that been implicated in human diseases or disorders (Additional file 2), including susceptibility to schizophrenia, diabetes, deafness, and mental retardation [38].

### Pleiotropic effects

Many of the *P*-element lines included in this screen have previously been examined for mutational effects on numbers of sensory bristles [30], resistance to starvation stress [31], olfactory behavior [33], 24-hour sleep [32] and locomotor reactivity (a startle response [34]). Mutational correlations (*r*_*M*_) between aggressive behavior and male abdominal bristle number (*n *= 160, *r*_*M *_= 0.09, *P *= 0.293), male sternopleural bristle number (*n *= 160, *r*_*M *_= 0.11, *P *= 0.183), olfactory avoidance behavior (*n *= 158, *r*_*M *_= 0.01, *P *= 0.94), starvation resistance (*n *= 88, *r*_*M *_= 0.09, *P *= 0.40), and 24-hour sleep (*n *= 28, *r*_*M *_= 0.20, *P *= 0.30) were not significantly different from zero. Mutational correlations could be non-significant if mutations specifically affect aggressive behavior, or if there are pleiotropic effects of mutations affecting aggression on other traits, but the effects are not in the same direction. It is the second explanation which is true – the mutations affecting aggressive behavior are highly pleiotropic, but mutations associated with increases (decreases) in aggressive behavior are not consistently associated with increases (decreases) of resistance to bristle number, starvation stress, olfactory behavior, or sleep (Table 1).

**Figure 3:**
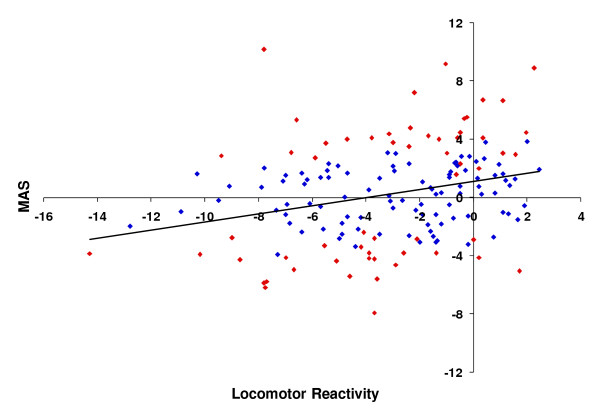
**Correlation between mean aggression score (MAS) and locomotor reactivity in *P*-element insertion lines**. Scores are given as a deviation from the control line. Data points in red represent lines with levels of aggressive behavior that are significantly different from the control.

The mutational correlation between aggressive behavior and locomotor startle response, although weak, was significantly different from zero and positive (*n *= 157, *r*_*M *_= 0.29, *P *= 0.0002; Figure 3). The mutational correlation for the subset of 58 lines with significantly increased and decreased levels of aggression and for which locomotor startle data was available was not significantly different from that estimated from all 157 lines (*r*_*M *_= 0.40, *P *= 0.0019). Overall, variation in locomotor startle response only explains 8% of the variation in aggressive behavior. This is largely attributable to a few insert lines with decreased levels of both aggression and duration of the locomotor startle response. However, many lines with increased levels of aggression had reduced locomotor startle responses.

### Analysis of P-element excision alleles

**Figure 4:**
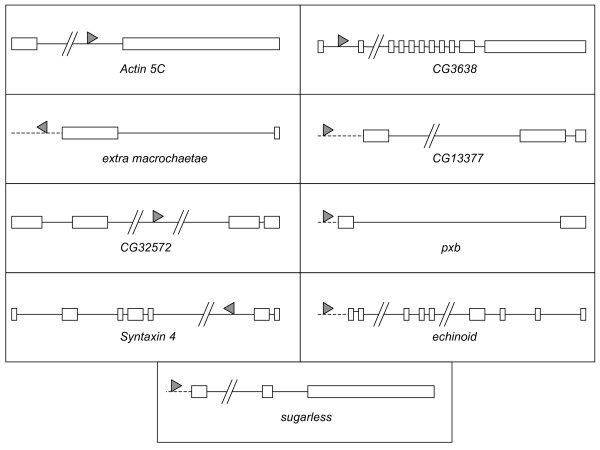
**Structure of nine genes in which mutations affect male aggressive behavior**. All genes are oriented 5' to 3', with boxes indicating exons and solid lines indicating introns. Dashed lines represent 5' putative promoter regions. Solid triangles indicate the location of the *P*-element insertion, with the direction of the triangle indicating the orientation of the insertion.

Nine lines were chosen for more detailed analysis that had large effects on aggressive behavior, and in which the *P*-element insertion site was located within the gene or in the presumed 5' regulatory region (Figure 4). The *P*-element insertions in *Actin 5C *(*Act5C*), *extra macrochaetae *(*emc*), *CG32572*, and *Syntaxin 4 *(*Syx4*) were associated with decreased levels of aggression; while *P*-element insertions in *pxb*, *echinoid *(*ed*), *sugarless *(*sgl*), *CG3638*, and *CG13377 *were associated with increased levels of aggression. We attempted to generate precise revertant alleles of each of these *P*-element tagged genes, in order to map the mutant phenotype to the *P*-element insertion. The revertant alleles were sequenced, and at least one precise revertant was identified for each line except *CG3638*. The effects of imprecise excision alleles were also evaluated for the lines in which no or only a single precise revertant allele was generated.

**Figure 5:**
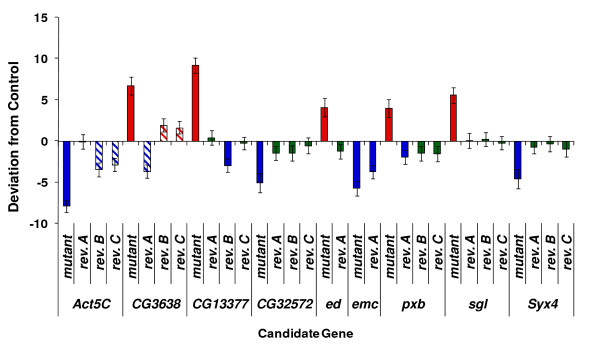
**Mean aggression scores of *P*-element mutations affecting aggressive behavior and revertant alleles**. The mean aggression score is given as the deviation from the contemporaneously tested control line for the mutant lines and up to three revertant alleles. Blue bars indicate significantly (*P *< 0.05) lower levels of aggression than the control; red bars indicate higher levels of aggression than the control; and green bars indicate no significant difference in mean aggression score from the control. Hatched bars indicate imprecise revertant alleles.

The aggressive behavior of the revertant alleles was quantified, and for seven of the nine lines the behavior of the precise excision alleles also reverted to control levels, thus mapping the mutant phenotype to the *P*-element insertion in the tagged gene (Figure 5). The exceptions were *emc *and *CG3638*. The *emc *precise revertant allele only showed partial phenotypic reversion. The behavior of one of the *CG3638 *imprecise revertants differed only marginally from that of the control (*P *= 0.04). The failure of the behavior of precise excision alleles to revert to the level of the control could indicate that the insertions of the *P*-elements in these loci do not cause the mutant phenotype. However, the observation of partial phenotypic reversion in conjunction with reduced levels of expression of *CG3638 *and *emc *in the respective mutant alleles is consistent with a complex mutation in the precise excision alleles; for example, local hopping of the *P*-elements elsewhere in *emc*.

### Analysis of gene expression

**Figure 6:**
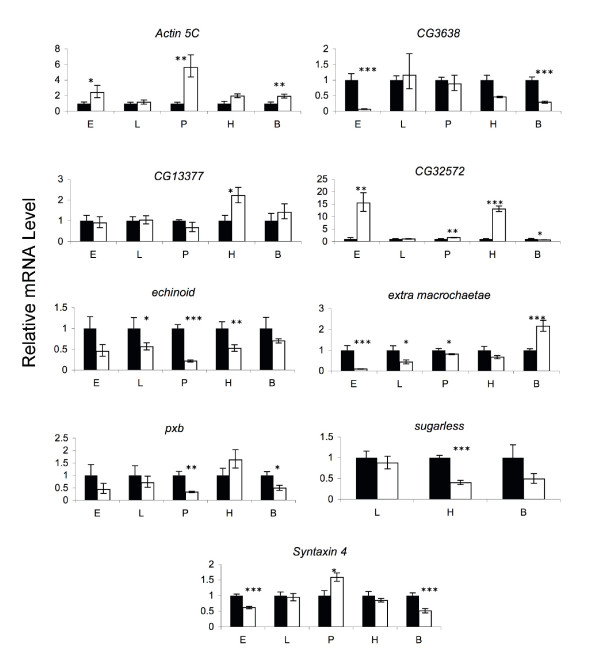
**Quantitative reverse transcription-PCR analysis of candidate genes affecting aggressive behavior**. Levels of mRNA for each gene (white bars) are depicted relative to the level in the co-isogenic control (black bars). mRNA levels were assessed at four developmental time periods: embryos aged 10–12 h after egg laying (E), third instar larvae (L), pupae (P), and adults (heads [H] and headless bodies [B]). Only larvae and adults could be obtained for *sgl *mutants. Standard errors were obtained using *Ct *values normalized to an internal control (*Gapdh1*). The significance of two-tailed Student's *t*-tests conducted on linearized *Ct *values are depicted by asterisks (*: *P *< 0.05; **: *P *< 0.01; ***: *P *< 0.001).

qPCR analyses were used to assess the effect of the *P*-element insertion on transcript levels of the tagged genes in the nine lines selected for further characterization. Since many of these genes have roles in development, expression was evaluated in embryos, larvae, pupae, and adults. The adult tissues were separated into heads and headless bodies (with the exception of the *sgl *mutant line, for which insufficient tissue was obtained to conduct qPCR on pupae or embryos, due to poor viability). All mutant lines were associated with alterations in transcript abundance in one or more developmental stages, confirming that the *P*-element insertions affect the expression of all tagged genes (Figure 6).

There was no consistent pattern of gene expression changes in mutations associated with decreased levels of aggression. *Act5C *and *CG32572 *mutants were associated with increased transcript levels – in embryos, pupae, and adult bodies for *Act5C*, and in embryos and adult heads for *CG32572*. Transcript levels in *emc *mutants were decreased throughout development, but increased in adult bodies. *Syx4 *mutants had reduced levels of gene expression in embryos and adult bodies, but increased expression in pupae. Mutations associated with increased aggression tended to have decreased levels of transcript abundance at one or more developmental stages. Gene expression was reduced in embryos and adult bodies of *CG3638 *mutants; in larvae, pupae, and adult heads of *ed *mutants; in pupae and adult bodies of *pxb *mutants; and in adult heads of *sgl *mutants. In contrast, there was an increase of transcript abundance in adult heads of *CG13377 *mutants.

This analysis shows that none of the mutations affecting aggressive behavior are transcriptional null alleles. The effects of all of the mutations on gene expression varied across development, and between adult heads and bodies. Depending on the developmental time point and/or adult tissue assessed,*CG3638*, *ed*, *pxb *and *sgl *are hypomorphic mutations; *Act5C *and *CG13377 *are hypermorphic mutations; and *CG32572*, *emc *and *Syx4 *are both hypomorphs and hypermorphs. All of the mutations showed significant differences in gene expression from the control in adults, but these differences were apparent in heads of only four of the mutations (*CG13377*, *CG32572*, *ed *and *sgl*). Further, many of the alterations in gene expression between mutant and control lines were of the order of two-fold or less. These results indicate that even subtle mutational effects on transcription can be associated with large changes in behavior. Because the effects of the mutations on gene expression in pre-adult stages are often much larger than observed for adults, we cannot rule out the possibility that changes in gene expression during development affect adult behavior [33].

The lack of a common pattern of gene expression differences among the mutations affecting increased and decreased levels of aggression suggests that there are multiple mechanisms by which this complex behavior can be altered. Finally, the observation that only four of the nine mutations show changes in gene expression in heads of adult flies indicates that only assessing changes in transcript abundance in heads of lines that are genetically divergent for behavioral traits will underestimate the number of transcripts associated with differences in the trait phenotype [26,47,48].

**Figure 7:**
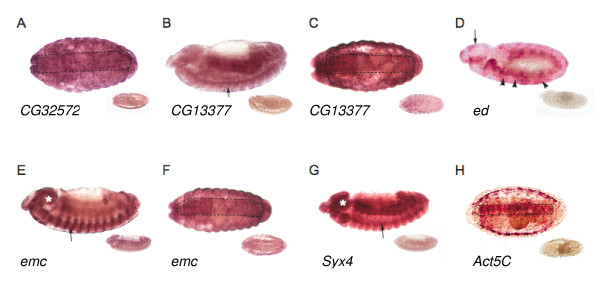
**Expression of candidate genes affecting aggressive behavior in the embryonic nervous system**. The insets show the sense control probes. **(A) ***CG32572*, stage 17, ventral view: expression in the ventral cord (highlighted with dotted line). **(B) ***CG13377*, stage 14, lateral view: expression in the ventral nerve cord (arrow). **(C) ***CG13377*, stage 17, ventral view: expression in the ventral nerve cord (highlighted with dotted line). **(D) ***ed*, stage 11, lateral view: expression in the procephalic neuroblasts (arrow) and the neuroblasts forming the ventral nerve cord (arrowheads). **(E) ***emc*, stage 14, lateral view: expression in the ventral nerve cord (arrow) and the brain (asterisk). **(F) ***emc*, stage 17, ventral view: expression in the ventral nerve cord (highlighted with dotted line). **(G) ***Syx4*, stage 13, lateral view: expression in the ventral nerve cord (arrow) and the brain (asterisk). **(H) ***Act5C*, stage 16, ventral view: expression in the ventral nerve cord (highlighted with dotted line).

We further characterized the patterns of expression of the nine *P*-element tagged genes affecting aggressive behavior in wild-type embryos. Consistent with previous results [49-53], *Act5C*, *emc *and *sgl *were expressed in multiple tissues, including the ventral nerve cord for *Act5C *and *emc*. *CG32572*, *CG13377*, *ed *and *Syx4 *were expressed in the central nervous system (Figure 7). Expression outside the nervous system was also observed for most of the genes (data not shown).

### Morphometric analysis of central brain neuropils

Mushroom bodies and the ellipsoid body are central brain neuroplils that have been previously implicated in *Drosophila *aggressive behavior. Disruption of mushroom body output results in near abolishment of aggression [11], and aberrant morphology of the mushroom bodies and ellipsoid body have been observed in hyper-aggressive mutants [28]. Therefore, we measured the length and width of the alpha and beta lobes of the mushroom bodies, and the surface area of the ellipsoid body, standardizing the values to overall brain size as a function of distance between peduncles (Table 2). There were significant quantitative changes in the length or width of one or both lobes of the mushroom bodies in all mutants except *emc*, further linking mushroom bodies and aggressive behavior. No significant differences in ellipsoid body area were observed for any of the mutations. The most frequently detected difference in mutants relative to control was an increase in the width of the alpha lobe. Only two of the mutations were associated with decreases in size: *Syx4 *mutants had shorter beta lobes than controls, and *sgl *mutants had shorter alpha lobes. Increases in beta lobe measurements were only observed for mutations associated with increased levels of aggression. However, there was no overall correlation between any of the quantitative measurements of brain morphology and aggressive behavior, consistent with previous studies [28,34] showing that there is no simple relationship between aggressive behavior and brain structure.

**Table 2:** Mushroom body measurements

	**Alpha lobes**		**Beta lobes**	
**Mutant**	**Length (SE)**	**Width (SE)**	**Length (SE)**	**Width (SE)**
*Canton S B*	6.03 (0.09)	0.6925 (0.0170)	4.28 (0.03)	0.7901 (0.0236)
*Act5C*	6.35 (0.13)*	0.6648 (0.0185)	4.18 (0.04)	0.7746 (0.0282)
*CG3638*	6.07 (0.13)	0.8372 (0.0323)***	4.26 (0.06)	0.8866 (0.0303)*
*CG13377*	5.99 (0.12)***	0.8036 (0.0222)	4.22 (0.04)	0.8273 (0.0342)
*CG32572*	6.56 (0.09)***	0.8218 (0.0169)***	4.23 (0.04)	0.8085 (0.0345)
*ed*	5.99 (0.16)	0.8330 (0.0299)***	4.09 (0.08)*	0.8683 (0.0389)
*emc*	6.23 (0.17)	0.7174 (0.0216)	4.29 (0.05)	0.8622 (0.0300)
*pxb*	6.12 (0.08)	0.8070 (0.0230)***	4.15 (0.05)*	0.8729 (0.0281)*
*sgl*	5.60 (0.13)**	0.7631 (0.0214)*	4.25 (0.07)	0.8263 (0.0312)
*Syx4*	5.94 (0.14)	0.7044 (0.0170)	4.13 (0.05)*	0.8504 (0.0194)

Measurements of the length and width of alpha and beta lobes (± SE) for *Canton S B *(the control line) and mutant lines associated with increased or decreased levels of aggression. Measurements are standardized to overall brain size. *: *P *< 0.05; **: *P *< 0.01; ***: *P *< 0.001.

**Figure 8:**
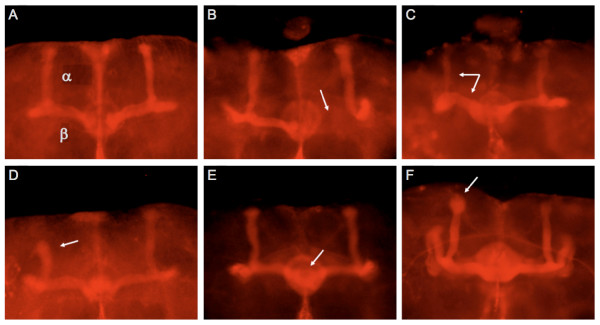
**Gross morphological defects in the mushroom bodies in mutations of candidate genes affecting aggressive behavior**. A-H: anti-fasciclin 2 staining of adult brains using the 1D4 monoclonal antibody. Defects are indicated by the white arrows. **(A) ***Canton S B *control line. α, alpha lobes of mushroom bodies; β, beta lobes of mushroom bodies. **(B) **Missing beta lobe in *CG32572 *mutation. **(C) **Misrouting of some of the alpha lobe axons leads to thicker beta lobes and thinner alpha lobes in *ed *mutation. **(D) **Shorter alpha lobe in *emc *mutation. **(E) **Overextension resulting in fusion of the beta lobes in *sgl *mutation. **(F) **Alpha lobe tip defect resulting in enlargement of the tip in *CG13377 *mutation.

In addition to quantitative alterations in brain morphology, we also observed qualitative morphological defects in both alpha and beta lobes for five of the mutant lines (Figure 8). One of ten brains examined for mutations of *CG32572*, *emc *and *CG13377 *had, respectively, a missing beta lobe, shorter alpha lobe, and an enlarged alpha lobe tip. Two of the ten *ed *mutant brains had qualitatively thicker beta lobes and thinner alpha lobes than the control, and three of the ten *sgl *brains had fused beta lobes. Although not completely penetrant, these defects were never observed in the control line.

### Candidate genes affecting aggressive behavior

None of the candidate genes identified in this screen have been previously implicated to affect aggressive behavior. Three of the nine candidate genes characterized in greater detail are computationally predicted genes. *CG13377 *is predicted to function in binding and metabolism [54]. RNAi-knockdown mutations of *CG3638 *display reduced phagocytic immune response to *Candida albicans *cells [55]. All that was previously known about *CG32572 *is that it is expressed in the testis [38]. *Act5C *is involved in ATP and protein binding [54], and also has roles in cytokinesis [56,57] and spermatogenesis [58]. *ed *has many developmental functions, including the negative regulation of neurogenesis [59], appendage formation [60], and negative regulation of epidermal growth factor signaling [61]. In adults, *ed *is expressed in ovaries, crop, and male accessory glands [38]. *emc *is also highly pleiotropic, and functions in peripheral nervous system [62], midgut [63], and spermatid development [64]. *pxb *mutants have been implicated in olfactory learning and memory [65] and in the *smoothened *signaling pathway [66]. *sgl*, like *pxb*, appears to be involved in *smoothened *signaling [67], metabolism [54], and biosynthesis [68,69]. *Syx4 *has been implicated in synaptic functions [70].

Given that we were able to map the behavioral mutant phenotype to the *P*-element insertion for seven of the nine mutations characterized in greater detail, and that all of the mutations affected gene expression of the tagged gene, we can predict that the majority of the remaining 48 *P*-element mutations associated with increased or decreased levels of aggression will also affect the genes into or near which they have inserted. Many of these genes are plausible candidates as they affect the development or the functioning of the nervous system (*Guanine nucleotide exchange factor GEF64C*, *NMDA receptor 1*, *schizo*, *tramtrack*, *Laminin A*, *longitudinals lacking*), and the effects of mutations in *neuralized *on aggressive behavior have been independently confirmed [28]. Many other genes affect other aspects of development, metabolism or basic cellular processes, or are computationally predicted – these loci would not have been detected had we concentrated on examining aggressive behavior for mutations in only 'plausible' candidate genes.

The general picture emerging from the analysis of quantitative effects of *de novo *mutations that have been induced in a defined isogenic background is that a large fraction of the genome can potentially affect most quantitative traits, including complex behaviors [30-34]. Consequently, we expect that most genes have pleiotropic effects on multiple traits, and indeed, 55 of the 59 mutations associated with a significant difference in aggressive behavior from the control line had pleiotropic effects on one (19 lines), two (22 lines), three (11 lines) or four (three lines) additional quantitative traits (Table 1). Further, different mutations in the same gene can have a different spectrum of pleiotropic effects [28,71], and the mutational effects on any one trait can be contingent on genetic background and the environment [28,33,72]. Given these complexities, an exhaustive mutational dissection of any complex behavior (or, indeed, any quantitative trait) is not feasible. However, the collection of over 70 mutations affecting aggressive behavior that have been generated in the same isogenic background (this study and [27]) are valuable molecular probes that can be used to gain insight into the key pathways and mechanisms affecting this trait using systems biology approaches [73].

## Conclusion

Aggressive behavior is important for survival and reproduction, and is near universal among animals. While the role of neurotransmitters in mediating and modulating levels of aggression is clear, little is known about other genes and pathways affecting aggression. Analysis of aggressive behavior in 170 *D. melanogaster P-*element mutant lines and their co-isogenic control lines revealed 59 mutations in 57 novel genes affecting aggression. More detailed characterization of nine of the mutations indicated that the *P*-element insertions affected the tagged genes. Most of the mutations had pleiotropic effects on other complex traits and on morphology of mushroom bodies, central brain neuroplils that have been previously implicated in *Drosophila *aggressive behavior.

## Abbreviations

AEL: after egg laying; ANOVA: analysis of variance; QTL: quantitative trait locus.

## Authors' contributions

ACE, TFCM, and PC designed the experiments and wrote the paper. ACE performed all behavioral measurements, bioinformatic analyses, molecular analyses of revertant alleles, and statistical analyses of behavioral and gene expression data. LZ performed the whole mount *in situ *hybridization analyses, immunohistochemistry and morphometric analyses of central brain neuropils, and statistical analysis of the morphometric measurements. AY created revertant alleles. All authors have read and approved the final manuscript.

## Supplementary Material

Additional file 1 **Mean aggression scores (MAS) of 170 *P{GT1} *insert lines**. This file shows the results of the screen of 170 *P*-element insert lines for alterations in aggressive behavior, the genes tagged by the *P*-elements, the mean aggression scores and effects, and gene ontology information. Click here for file

Additional file 2 **Human diseases associated with *P{GT1} *insertions in *Drosophila *genes with aberrant aggressive behavior**. This file shows the *P*-element insert lines with altered aggressive behavior, their mean aggression scores, and diseases in which the human orthologues of the tagged genes have been implicated. Click here for file
